# Supplementary material for: Bypassing Emergency Service: Decoding the Drivers of Self-Referral During Acute Myocardial Infarction on Rural Areas in Sachsen-Anhalt, Germany
Source: Healthcare (Basel). 2024 Nov 9;12(22):2234. doi: 10.3390/healthcare12222234 (PMC11593902; doi:10.3390/healthcare12222234)
Supplement: Supplementary file 1 [file healthcare-12-02234-s001.zip › healthcare-3175830-supplementary.pdf]

Table S1. Sample Characteristics, overall and by mode of transportation				
	Mode of Transport			
**Characteristic**	**Emergency Service**; [95% CI][12] **n= 2755**	**family Physician**; [95% CI][12] **n=570**	**Self-referral**; [95% CI][12] **n=719**	**Overall**; [95% CI][12] **n=4044**
**Sex**				
Male	64.1%; [62.2, 65.9]	65.6%; [61.5, 69.5]	69.5%; [66.0, 72.9]	65.3%; [63.8, 66.7]
Female	35.9%; [34.1, 37.8]	34.4%; [30.5, 38.5]	30.5%; [27.1, 34.0]	34.7%; [33.3, 36.2]
**Age categories**				
25-49	7.3%; [6.4, 8.4]	7.9%; [5.9, 10.5]	13.2%; [10.9, 16.0]	8.5%; [7.6, 9.4]
50-59	16.3%; [15.0, 17.8]	17.9%; [14.9, 21.3]	22.5%; [19.6, 25.8]	17.7%; [16.5, 18.9]
60-69	20.6%; [19.1, 22.2]	19.6%; [16.5, 23.2]	23.5%; [20.5, 26.8]	21.0%; [19.8, 22.3]
70-79	28.8%; [27.1, 30.5]	28.6%; [25.0, 32.5]	26.1%; [23.0, 29.6]	28.3%; [26.9, 29.7]
80+	26.9%; [25.3, 28.6]	26.0%; [22.5, 29.8]	14.6%; [12.1, 17.4]	24.6%; [23.3, 26.0]
**Region**				
Halle (Urban)	54.0%; [52.1, 55.9]	43.0%; [38.9, 47.2]	32.7%; [29.3, 36.3]	48.7%; [47.1, 50.2]
Altmark (Rural)	46.0%; [44.1, 47.9]	57.0%; [52.8, 61.1]	67.3%; [63.7, 70.7]	51.3%; [49.8, 52.9]
**Arterial Hypertension**	85.5%; [84.1, 86.8]	86.3%; [83.2, 89.0]	82.6%; [79.6, 85.3]	85.1%; [83.9, 86.2]
**Hypercholesterolemia**	50.6%; [48.7, 52.4]	59.1%; [55.0, 63.2]	56.7%; [53.0, 60.4]	52.9%; [51.3, 54.4]
**Diabetes**	34.9%; [33.1, 36.7]	42.8%; [38.7, 47.0]	31.6%; [28.2, 35.1]	35.4%; [33.9, 36.9]

**BMI**				
1) Normal weight or less	27.3%; [25.6, 29.0]	25.6%; [22.1, 29.4]	23.2%; [20.2, 26.5]	26.3%; [25.0, 27.7]
2) Overweight	40.6%; [38.8, 42.5]	40.0%; [36.0, 44.2]	42.0%; [38.4, 45.7]	40.8%; [39.3, 42.3]
3) Obesity I	21.8%; [20.3, 23.4]	22.6%; [19.3, 26.3]	24.5%; [21.4, 27.8]	22.4%; [21.1, 23.7]
4) Obesity II	6.7%; [5.8, 7.7]	8.8%; [6.6, 11.5]	7.2%; [5.5, 9.4]	7.1%; [6.3, 7.9]
5) Obesity III	3.6%; [2.9, 4.3]	3.0%; [1.8, 4.8]	3.1%; [2.0, 4.7]	3.4%; [2.9, 4.0]

[1]%

[2]CI = Confidence Interval
